# NITRIC OXIDE SYNTHASE DYSFUNCTION UNDERLIES CEREBROVASCULAR DEFICITS IN A MOUSE MODEL OF TAUOPATHY

**DOI:** 10.1093/geroni/igz038.346

**Published:** 2019-11-08

**Authors:** Candice E Van Skike, Stacy A Hussong, Andy Banh, Veronica Galvan

**Affiliations:** 1 Barshop Institute - UT Health San Antonio, San Antonio, Texas, United States; 2 University of Texas Health Science Center at San Antonio, San Antonio, Texas, United States; 3 Barshop Institute of Longevity and Aging Studies - UT Health San Antonio, San Antonio, Texas, United States; 4 Barshop Institute for Longevity and Aging Studies, San Antonio, Texas, United States

## Abstract

We recently identified pathogenic soluble aggregated tau (tau oligomers) in the cerebral microvasculature of human patients with tauopathies, including Alzheimer’s disease (AD). The functional consequences of cerebrovascular tau accumulation are not yet understood. The aim of the present study was to determine whether pathogenic tau accumulation leads to cerebrovascular dysfunction. To this end, we measured neurovascular coupling (NVC), a highly regulated process that synchronizes cerebral blood flow to neuronal activation, using the PS19(P301S) mouse model of tauopathy. The change in cerebral blood flow evoked by whisker stimulation was measured using Laser Doppler flowmetry in PS19 and wildtype control mice and the functional contribution of neuronal and endothelial nitric oxide synthase (nNOS and eNOS, respectively) was calculated. Vascular reactivity was assessed using topical acetylcholine to evoke endothelium-dependent vasodilation. To assess the direct impact of pathogenic tau on cell-specific NOS function, we treated N2a neuroblastoma cells or mouse brain vascular endothelial cells with soluble tau aggregates and measured activity of nNOS and eNOS. Our data indicate isolated overexpression of mutant tau impairs NVC responses, and this deficit is mediated by a reduction in nNOS activity in vivo. Further, our studies suggest tauopathy also impairs endothelium-dependent vasoreactivity in the cortex. Additionally, soluble tau aggregates inhibit the phosphorylation of NOS in primary cultured cells. Therefore, inhibition of NOS phosphorylation by pathogenic soluble tau aggregates may underlie cerebrovascular dysfunction in tauopathies. Thus, therapeutic modulation of pathogenic tau may mitigate brain microvascular deficits, which occur prior to clinical onset in Alzheimer’s disease and potentially other tauopathies.

